# Increasing Effectiveness and Equity in Strengthening Health Research Capacity Using Data and Metrics: Recent Advances of the ESSENCE Mechanism

**DOI:** 10.5334/aogh.3948

**Published:** 2023-06-02

**Authors:** Peter H. Kilmarx, Thabi Maitin, Taghreed Adam, Garry Aslanyan, Michael Cheetham, Janelle Cruz, Martin Eigbike, Oumar Gaye, Catherine M. Jones, Linda Kupfer, John Lindo, Rhona Mijumbi, Jean B. Nachega, Jamie Bay Nishi, Irini Pantelidou, Malabika Sarker, Soumya Swaminathan

**Affiliations:** 1 Fogarty International Center, U.S. National Institutes of Health, Bethesda, Maryland, USA; 2 South African Medical Research Council, Parow Valley, Cape Town, Western Cape, South Africa; 3 Research for Health Department, World Health Organization, Geneva, Switzerland; 4 Special Programme for Research and Training in Tropical Diseases (TDR), World Health Organization, Geneva, Switzerland; 5 Office of Portfolio Analysis, U.S. National Institutes of Health, Bethesda, Maryland, USA; 6 Gawani Africa, Abuja, Nigeria; 7 Cheikh Anta Diop University, Dakar, Senegal; 8 Department of Health Policy, London School of Economics and Political Science, London, UK; 9 University of the West Indies, Kingston, Jamaica; 10 Makerere University College of Health Sciences, Kampala, Uganda; 11 Department of Epidemiology School of Public Health and Center for Global Health, University of Pittsburgh, Pittsburgh, Pennsylvania, USA; 12 Department of Medicine, Stellenbosch University Faculty of Medicine and Health Sciences, Cape Town, South Africa; 13 Department of Epidemiology, Johns Hopkins Bloomberg School of Public Health, Baltimore, Maryland, USA; 14 Department of International Health, Johns Hopkins Bloomberg School of Public Health, Baltimore, Maryland, USA; 15 Global Health Technologies Coalition, Washington DC, USA; 16 Wellcome Trust, London, UK; 17 James P. Grant School of Public Health, BRAC University, Dhaka, Bangladesh; 18 Science Division, World Health Organization, Geneva, Switzerland

**Keywords:** research capacity, capacity strengthening, metrics, funder coordination

## Abstract

**Background:**

The ESSENCE on Health Research initiative established a Working Group on Review of Investments in 2018 to improve coordination and collaboration among funders of health research capacity strengthening. The Working Group comprises more than a dozen ESSENCE members, including diverse representation by geography, country income level, the public sector, and philanthropy.

**Objective:**

The overall goal of the Working Group is increased research on national health priorities as well as improved pandemic preparedness, and, ultimately, fewer countries with very limited research capacity.

**Methods:**

We developed a basic set of metrics for national health research capacity, assessed different models of coordination and collaboration, took a deeper dive into eight countries to characterize their national research capacity, and began to identify opportunities to better coordinate our investments. In this article, we summarize the presentations, discussions, and outcomes of our second annual (virtual) meeting, which had more than 100 participants representing funders, researchers, and other stakeholders from higher- and lower-income countries worldwide.

**Findings and conclusions:**

Presentations on the first day included the keynote speaker, Dr. Soumya Swaminathan, chief scientist of the World Health Organization (WHO), and updates on data and metrics for research capacity, which are critical to establish targets, road maps, and budgets. The second day focused on improving collaboration and coordination among funders and other stakeholders, the potential return on investment for health research, ongoing work to increase coordination at the country level, and examples of research capacity strengthening efforts in diverse health research areas from around the world. We concluded that an intentional data- and metric-driven approach to health research capacity strengthening, emphasizing coordination among funders, local leadership, and equitable partnerships and allocation of resources, will enhance the health systems of resource-poor countries as well as the world’s pandemic preparedness.

## Introduction and Background

The ESSENCE on Health Research initiative is a forum of funders of health research capacity strengthening in low- and middle-income countries (LMICs) coordinated by the Special Programme for Research and Training in Tropical Diseases (TDR) based at the World Health Organization (WHO). The ESSENCE Working Group on Review of Investments (hereafter called the Working Group) was formed to improve coordination and collaboration among funders of health research capacity strengthening following a report sponsored by the World Bank and the Coalition for Epidemic Preparedness Innovations titled *Money and Microbes*, which was launched at the World Health Assembly in 2018 [1]. The report’s key recommendation was that clinical research capacity should be considered an essential element of pandemic preparedness. It also recommended that ESSENCE, as a forum of funders of health research capacity building [2], should articulate a mechanism to review their investments.

The Working Group was established with more than a dozen ESSENCE members, including diverse representation by geography, country income level, the public sector, and philanthropy. In the first year, the framework for the mechanism was established whereby funders would use data from the WHO Global Observatory on Health Research and Development (R&D) [3] and from the National Institutes of Health (NIH) World RePORT [4] to help guide their activities for the greatest impact [5]. Funders are encouraged to coordinate activities with each other to increase their effectiveness and equity and decrease duplication of effort.

The overall goal of the Working Group is increased research on national health priorities as well as improved pandemic preparedness and, ultimately, fewer countries with very limited research capacity. By 2020, the Working Group had developed a basic set of metrics for national health research capacity. We also assessed different models of coordination and collaboration, and we took a deeper dive into eight countries to characterize their national research capacity and to begin to identify opportunities to better coordinate our investments.

In this article, we summarize the presentations, discussions, and outcomes of our November 1–2, 2021, virtual meeting, which had more than 100 participants representing funders, researchers, and other stakeholders from higher- and lower-income countries worldwide. The first keynote speaker, Dr. Soumya Swaminathan, shared her vision for improving coordination and strengthening capacity for research for health from her perspective as a researcher, former director general of the Indian Council of Medical Research, and current chief scientist of the WHO. Other presentations on the first day included updates on data and metrics for research capacity, which are critical to establish targets, road maps, and budgets.

The second day focused on improving collaboration and coordination among funders and other stakeholders. The keynote speaker, Dr. Jean Nachega, shared his perspectives from decades of experience connecting African and global researchers and funding organizations. Others spoke about the potential return on investment for health research, ongoing work to increase coordination at the country level, and examples of capacity strengthening efforts in diverse health research areas from around the world.

## WHO Vision for Coordination and Strengthening Research Capacity

Despite substantial global investments in COVID-19 research in 2020 and 2021, there was a disappointing lack of a coordinated response from existing clinical trials networks. A WHO scientific gathering in February 2020 created a road map for the development of COVID-19 diagnostics, vaccines, and therapeutics, resulting in the *WHO R&D Blueprint: Novel Coronavirus* [6]. But, with some exceptions, there was not subsequent effective collaboration and coordination across research funders to establish large multicountry studies using common protocols and common frameworks. WHO responded by establishing an international collaboration to identify lifesaving treatments for COVID-19 [7]. The pandemic has led to scientific gains, such as advances made in data exchange and use of genomic sequence data to track the evolution and spread of the virus. Funders of health research capacity strengthening should take each of these lessons learned into account, build on this progress, and continue to ensure global representation in these discussions.

## Global Health Security Agenda R&D Taskforce

No global framework currently exists for assessing and strengthening the capacity of countries to develop, approve, manufacture, and deploy vaccines, therapeutics, diagnostics, and other medical countermeasures. R&D is not explicitly integrated into the WHO International Health Regulations, Joint External Evaluations, or the Global Health Security Agenda (GHSA), which is a global effort to strengthen the world’s ability to prevent, detect, and respond to infectious disease threats. In the absence of a global framework for R&D capacity strengthening, donors and implementing countries have been slow to prioritize this essential work for strengthening epidemic preparedness.

To help fill this gap in the global norms for pandemic preparedness, GHSA approved a proposal to launch an R&D Task Force in the summer of 2021 [8]. The R&D Task Force is developing tools to help GHSA countries and donors make a clear case for the link between R&D capacity building and health security targets, to set clear road maps for strengthening R&D capacity building, and to hold dialogues that help identify and highlight key bottlenecks for decision makers. There is an opportunity to link the efforts of ESSENCE with the GHSA to strengthen assessments of country and regional research capacity, identify gaps, and highlight indicators and metrics that can track progress for GHSA members as local R&D capabilities are enhanced.

## Metrics and Indicators: Sources, Analysis, and Critique

### ESSENCE Metrics

The ESSENCE metrics of national health research capacity developed in 2020 [9] were updated for 2021. Analyses were conducted to better understand research capacity and correlates with other country characteristics [10]. All countries with a population greater than 100,000 were included (N = 180 countries). Three widely available indicators for each country were used, averaging the results from 2018–2020: (1) number of clinical trials in the WHO International Clinical Trials Registry Platform (ICTRP) [11]; (2) number of records of research activities in the NIH World RePORT database [4]; and (3) number of publications with an author affiliation in the country in Scopus [12]. Country rank percentiles for each indicator were averaged to create an overall metric of health research capacity, recognizing that this is a very basic metric and that more information from country representatives is necessary to determine national research priorities and specific barriers and facilitators.

Univariate analyses of the indicators of health research capacity showed highly skewed distributions with differences among countries (N = 180) by geographic region and by income group. Results of Kendall’s Tau test showed strongly positive correlations among the three indicators, with values ranging from 0.60 to 0.78. Higher absolute values of tau (0–1) indicate a higher correlation. This suggests good internal consistency and potential reliability of this set of indicators. We also conducted correlation tests between the aggregate metric and select sociodemographic country indicators and found moderate to strong correlations with overall gross domestic product (GDP) (tau = 0.78), total population (tau = 0.56), disability-adjusted life years per capita (tau = –0.34), Human Development Index (tau = 0.31), and GDP per capita (tau = 0.25).

These results confirmed our impression that larger, higher-income countries tended to have greater research capacity; however, the results also show that many smaller, lower-income, and less-developed countries also have good research capacity. There may be opportunities to apply lessons learned from these outliers for other countries to follow. The analysis further demonstrated that relatively simple metrics for assessing country-level health research capacity can be created using publicly available data. These metrics are intended for use by funders, national health authorities, researchers, and other stakeholders to plan and implement effective and equitable initiatives to strengthen health research capacity, for example, by focusing some portion of available resources to those countries with the greatest need.

### WHO Global Observatory on Health R&D

The WHO Global Observatory on Health R&D [3] is a comprehensive source of information and analyses on global health R&D and a critical element of the ESSENCE Mechanism. Working with the WHO’s regional offices, the observatory team is establishing a global set of harmonized and agreed upon core indicators to assess national health research systems, with the goal of regularly reporting on this information. The purpose of this global set of indicators is to provide a high-level picture of national health research systems using comparable measures across countries. The measurements generated will be accessible and updated on a regular basis.

### NIH World RePORT

World RePORT is an interactive, open-access database hosted by NIH that maps research investments from some of the world’s largest biomedical funding organizations [4] and another key component of the ESSENCE Mechanism. Comprising over 700,000 project records for research in over 26,000 institutions in 187 countries from 14 funders, it is designed to facilitate funding analysis and visualization of global biomedical research networks.

### Caveats on the use of metrics and the case for building research capacity in Africa

When decision makers can evaluate and measure a country’s performance in health research, this may facilitate planning and allocation of resources. However, health research systems are complex, and attempts to simplify their measurement risk leading to inadequate representations. Metrics are decidedly political, with choices made on what to count in the first place, where to count it, and how to use or aggregate data [13]. For instance, the number of patents, a common measure for innovation performance, does not distinguish between the usefulness or quality represented by the patent [14]. Mapping of available data on indicators of health research and development in Africa shows a nuanced picture, with mixed performance across indicators within countries and important gaps due to missing data [15]. Importantly, several health research decision makers in Africa have underlined how the use of facile global indicators is a form of power that can impede the appreciation of local, context-specific knowledge [16].

These critiques of metrics should be situated within a broader systems perspective, which researchers from the London School of Economics explored qualitatively with African partners, drawing from Pang’s [17] definition of a national health research system:

“The people, institutions, and activities whose primary purpose is to generate high-quality knowledge that can be used to promote, restore, and/or maintain the health status of populations.”

**Figure 1:**
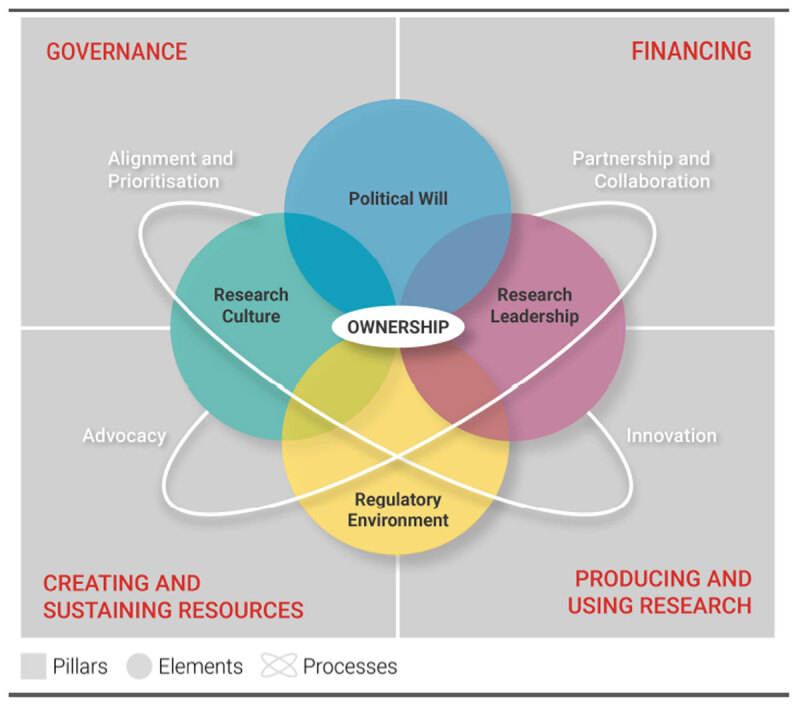
Empirically informed conceptual framework of national health research systems from a systems perspective. From: Jones CM, Ankotche A, Canner E, et al. *Strengthening National Health Research Systems in Africa: Lessons and Insights from across the Continent*. LSE Health; 2021. p. 22. Available at: https://doi.org/10.6084/m9.figshare.14039807.

They used data from 189 qualitative interviews, and from these findings, an empirically informed framework was created, illustrating strengthening national health research systems from a systems perspective (Figure 1) [18]. The four pillars—governance, financing, creating and sustaining resources, and producing and using knowledge—are represented as core functions, as proposed by the African barometer, which is used by WHO Africa to assess progress and inform development of national health research systems in Africa [19,20]. Various combinations of the processes and elements come through clearly as factors that influence the development and trajectories of national health research systems [15,18,21]. Research leadership, advocacy, and the regulatory environment frequently worked together in long-term improvements for the governance for health research [22].

## Best Practices and Lessons Learned Building Research Capacity in Africa

Over the past few decades, the amount of research carried out in the WHO Africa region has increased substantially, whether conducted by Africans alone or in collaboration with partners from high-income countries [23]. However, the increase in research outputs has not been equally shared among African countries. English-speaking countries produce more scientific articles compared to countries speaking French, Portuguese, or Arabic [23]. Research productivity in Africa also increased by 96% per US$1 increase in a country’s GDP, and for every single increase in the number of postgraduate training institutions in a country, research productivity increased by 298% [23].

Research capacity building initiatives on the Africa continent include the Southern Africa Consortium for Research Excellence (SACORE) [24], funded under the Wellcome Trust African Institutions Initiative, and the Medical Education Partnership Initiative (MEPI) [25], which provided grants to 13 medical schools in 12 sub-Saharan African countries supported by the U.S. President’s Emergency Plan for AIDS Relief (PEPFAR) and NIH. Analysis of such programs demonstrates clear achievements as well as key challenges, such as long-term sustainability due to dependence on foreign funding. Selected best research capacity building practices included (1) the establishment of research support centers; (2) South–South partnerships, collaborations, and networking; (3) training of fellows in scientific manuscript writing in English, research grant proposal writing, and training in research and laboratory skills; (4) triangular mentorship networks with local-, regional-, and international-based advisers; (5) annual scientific meetings that provide a forum for scientists to present their research and network; and (6) diversification of sources of research funding with less dependency on international donors and more reliance on local government-funded research (Table 1). Increased investment in health research from African governments would allow them to take charge of the research agenda and sustain existing capacity building efforts. Reducing inequities in health research capacity between countries and African subregions is another important goal.

**Table 1:** Critical elements of sustainable health research capacity strengthening in low- and middle-income countries (LMICs).


1	Training diverse cadres in research methodology and writing scientific manuscript and grant proposals

2	Establishing and maintaining infrastructure and core capacities with research clinics, laboratory testing, biorepositories, data management, biostatistics, research ethics review, and research regulatory capacity

3	Research support centers, including grant and financial management

4	Equitable North–South and South–South partnerships, collaborations, and networking

5	Mentorship networks with local, regional, and international advisers for continuous development opportunities and support

6	Regular scientific meetings for scientists at all career stages to present research and network

7	Diversification of sources of research funding and increasing LMIC government and private sector funding

8	Country-led research agendas and demand for research to inform evidence-based policies

9	Community engagement with members of research populations, policy makers, and other stakeholders

10	Data- and milestone-driven monitoring and evaluation frameworks for research capacity


## Examples of Health Research Capacity Strengthening

The following are three examples of health research capacity strengthening and funding coordination from diverse regional or national perspectives in lower-resource settings.

### West Indies

The University of the West Indies (UWI) recognized the need for capacity building in health research and took advantage of an agreement with the State University of New York (SUNY), with its 64 campuses, to collaborate on health-related research. In collaboration with the SUNY Global Health Institute, a Health Research Taskforce was established in 2015 under the SUNY-UWI Center for Leadership and Sustainable Development with the goal of advancing public health in the Caribbean region through collaborative research and education initiatives among faculty and students at SUNY and UWI. The taskforce transitioned to a consortium in 2019 and is now a dynamic, integrated matrix that is addressing health research challenges and planning innovative approaches that use shared management through senior leadership partnerships.

A needs assessment was conducted by a team from the SUNY Global Health Institute and the Mona campus (UWI) that identified the following health research priority areas: emerging infectious diseases; oncology; cardiovascular, hepatic, and renal diseases; diabetes, metabolic, and digestive diseases; and behavioral and mental health. This approach has been highly productive and led to the creation of an array of health research focus areas. As an example, the emerging infectious disease theme was developed in response to the wave of epidemics of dengue, Chikungunya, and Zika infections that affected the Caribbean and was in an advanced stage when SARS-CoV-2 emerged in 2019. Significant capacity building occurred through successful funding from the NIH Fogarty International Center to support a Global Infectious Diseases Research Training Program (GIDRTP). Through this program, collaborative mentoring from SUNY and UWI led the training of scholars at the master’s and doctoral levels in virology, immunology, epidemiology, drug development, viral surveillance, and pathogen discovery. In tandem, two Affiliate Centers of Excellence of the Global Virus Network were established at the Mona (Jamaica) and St. Augustine (Trinidad and Tobago) campuses of the UWI that provided additional support to the training. The consortium also established working relationships with partners at Rush University, where the GIDRTP immunology training core was established, and the Abbott Pandemic Defense Coalition, with a focus on viral surveillance and pathogen discovery. This successful research training program was replicated, and new teams have engaged to submit grant applications in pelvic oncology and noncommunicable diseases associated with the oral microbiome.

### West and Central Africa

Several capacity building initiatives are ongoing in West and Central Africa, where substantial improvements were achieved through several research programs. The Malaria Research Capacity Development in West and Central Africa (MARCAD) is one of these programs supported by the Developing Excellence in Leadership, Training and Science in Africa (DELTAS Africa), which is a major initiative to strengthen health research capacity in Africa. The objectives of MARCAD are to support high-quality research in malaria and neglected tropical diseases (NTDs), to develop career pathways for MARCAD fellows, to strengthen management and research environments, to develop a community and public engagement strategy, and to deliver evidence to support programs to control malaria and NTDs.

One of the key aspects of the MARCAD program is in research leadership. Master’s degree students, PhDs and postdoctoral fellows, and midcareer researchers are recruited in partner institutions, taking into account gender and other diversity criteria. Supervision and mentorship from leading scientists are provided to the fellows, and courses and workshops are organized on topics that include malaria elimination, leadership, biostatistics, grant writing, data management, risk management, communication, and scientific diplomacy. Another critical aspect of the program is related to improving research management and infrastructure by providing financial support to partner institutions. Collaboration and networking with other research institutions in the subregion are also essential to share experiences and research data. Finally, community engagement with different stakeholders, policy makers, and communication specialists has been established to support the MARCAD program.

There have been many success stories. The evidence base has been strengthened for community case management of malaria, national malaria control programs are delivering proven-effective strategies for seasonal malaria chemoprevention and targeted mass drug administration, and significant decreases in malaria cases and deaths have been documented in Senegal and the Gambia. Other successes include academia, nongovernmental organization, and national malaria control program positions that have been obtained by fellows and new research grants that have been awarded to the partner institutions.

The funding sources that have helped build capacity for research for health in West and Central Africa are quite diverse. Building a regional research consortium linking anglophone and francophone stakeholders and demonstrating strong linkages to health policy, practice, and outcomes are the main indicators of success in the MARCAD and DELTAS program. The establishment of a supportive research environment with a high consideration of research career paths has been essential. Continued funding with an increasing proportion of domestic funding will be necessary to sustain these successes.

### Bangladesh

Bangladesh is known for conducting health-related research and publishing in peer-reviewed scientific journals in the region. However, development of research capacity, defined as a country’s ability to produce, debate, and use research knowledge and products relevant to the country’s needs [26], is relatively weak.

The common practice in Bangladesh is to implement individual-focused sporadic capacity development initiatives through training and scholarships organized by individual institutions. These training programs are mainly limited to a wide range of short courses and a few modules in a master’s degree program. Developing the capacity of research departments in universities, research institutes, think tanks, and others to fund, manage, and sustain themselves and interaction with researchers has been less common, with the notable exception of icddr,b (formerly known as the International Centre for Diarrhoeal Disease Research, Bangladesh), a research organization that has impacted both local and global health policy and practice. The importance of creating a conducive environment for research capacity within institutions is a crucial stepping stone to sustainability.

Bangladesh is investing significantly to create an enabling environment for conducting research. However, there has not been a robust public national-level initiative on research capacity development. The Ministry of Health of Bangladesh has allocated a substantial fund for conducting research in the health sector. However, no policy or guideline for systematic research capacity development at the national level currently exists.

The only relevant publicly available document developed by the Bangladeshi government in the health sector is a research strategy [27] published in 2011. This document elaborated a framework on research capacity development that was never executed. Since then, no further initiative has been taken, and the Health Nutrition Population Strategic Investment Plan (HNPSIP) of 2016–2021 did not include any plan or framework for research capacity development [28]. A recent study conducted by a public postgraduation institute in Bangladesh indicated the major challenge for conducting research is the absence of a training program for skill development [29].

There is, therefore, a need for the national research capacity building initiative that is being led by the Ministry of Health in collaboration with the local research institutes like icddr,b; the National Institute of Preventive and Social Medicine (NIPSOM); and the BRAC James P Grant School of Public Health. The focus of the strategic plan must include infrastructural upgrades, creating research administration units, training of trainers, and execution plans. Each institute should prepare an annual research capacity development plan with budget allocations. A monitoring and evaluation framework in the strategic plan will allow assessment of the outcome of such an initiative. Integrating the strategic plan in the five-year HNPSIP would help ensure a successful outcome.

## Summary and Conclusions

The capacity to conduct research for health is an important element of national health systems and of global pandemic preparedness. Each country should be able to conduct research to address its national priorities, and the ability to conduct R&D in any country is a global public good in the event of an infectious disease outbreak. The ESSENCE Working Group on Review of Investments brings together leading funders of health research capacity strengthening in LMICs to use data and metrics to increase the effectiveness and equity of these efforts. The COVID-19 pandemic has revealed shortcomings in the world’s health research systems but also some notable strengths in many LMICs, as well as many lessons learned for future capacity strengthening and coordination. Data from the WHO Global Observatory on Health R&D and NIH World RePORT are being used for a basic metric of health research capacity, which helps to indicate where needs are the greatest and where focused efforts are required. GHSA and other authorities on pandemic preparedness are also adopting the use of metrics of research capacity, but more work is needed to develop specific indicators of research preparedness beyond the basic metric.

Experience has shown considerable progress in capacity strengthening in Africa and other resource-poor areas. Some of the best practices include the establishment of research support centers with grant management capabilities, South–South partnering, comprehensive training of early career researchers, and diversification of research funding sources, including local resource mobilization (Table 1). Care is needed in measuring and evaluating national health research capacity. The national interest in promoting a country’s population health should be paramount. Local research leadership, advocacy, and a conducive regulatory environment are needed for long-term improvements. In collaborations with funders, a variety of stakeholders needs to be included, and local leadership and ownership need to be established from the beginning. Diverse examples of research capacity strengthening in the West Indies, West and Central Africa, and South Asia further emphasize the importance of local leadership and ownership of robust national health research systems with regional and global partnership and support.

An intentional data- and metric-driven approach to health research capacity strengthening, emphasizing coordination among funders, local leadership, and equitable partnerships and allocation of resources, will enhance the health systems of resource-poor countries as well as the world’s pandemic preparedness.

## Disclaimer

The findings and conclusions in this paper are those of the authors and do not necessarily represent the views of their institutions.

## Data Accessibility Statement

Cited data available at https://tdr.who.int/docs/librariesprovider10/meeting-reports/appendix-list-of-all-countries-in-descending-order-by-aggregate-measure-of-national-health-research-capacity.xlsx?sfvrsn=3005a2d6_9&ua=1.
